# The effects of COVID-19 stay-at-home orders on physical activity of people with obesity

**DOI:** 10.31744/einstein_journal/2022AO6727

**Published:** 2022-04-13

**Authors:** Wagner Luiz do Prado, Mara Cristina Lofrano-Prado, Diego Giulliano Destro Christofaro, Carla Caroliny de Almeida Santana, Gabriel Grizzo Cucato, Matthew Jackson, Grace Shumate, Sarah Shumate, Marilia de Almeida Correia, João Paulo Botero, Raphael Mendes Ritti-Dias

**Affiliations:** 1 California State University San Bernardino San Bernardino CA United States California State University San Bernardino, San Bernardino, CA, United States.; 2 Universidade Estadual Paulista “Júlio de Mesquita Filho” Presidente Prudente SP Brazil Universidade Estadual Paulista “Júlio de Mesquita Filho”, Presidente Prudente, SP, Brazil.; 3 Universidade Federal Rural de Pernambuco Recife PE Brazil Universidade Federal Rural de Pernambuco, Recife, PE, Brazil.; 4 Northumbria University Newcastle United Kingdom Northumbria University, Newcastle, United Kingdom.; 5 Universidade Nove de Julho São Paulo SP Brazil Universidade Nove de Julho, São Paulo, SP, Brazil.; 6 Universidade Federal de São Paulo Santos SP Brazil Universidade Federal de São Paulo, Santos, SP, Brazil.

**Keywords:** COVID-19, Coronavirus infections, Overweight, Adult, Health, Exercise, Sedentary behavior

## Abstract

**Objective:**

To verify the association of changes on physical activity levels during coronavirus disease 2019 (COVID-19) outbreak of individuals with normal weight and overweight/obesity, and the influence of sex in this relationship.

**Methods:**

This cross-sectional study (survey research) was conducted in Brazil between May 5 and May 17, 2020. Participants (n=1,828 / 1,062 women >18 years) were invited through social media to answer a structured questionnaire via Google Forms. The online assessment included self-reported responses to questions on physical activity, overall health, weight, and height. Binary logistic regression analyzed the relationship between overweight/obesity (body mass index ≥25kg/m^2^), the impact of COVID-19 on physical activity level, and the influence of sex.

**Results:**

Compared to normal weight people, those with overweight/obesity practice less moderate to vigorous physical activity (p<0.001). There were associations between women and men with overweight/obesity and the impact of COVID-19 on the physical activity practice compared to normal weight people, adjusted by age, education level, social isolation, and previous physical activity level (p<0.017).

**Conclusion:**

The study found an association of weight and changes in physical activity levels. Individuals with overweight/obesity were more likely to have a lower physical activity level during COVID-19 pandemic, regardless of sex.

## INTRODUCTION

Coronavirus disease 2019 (COVID-19) is caused by severe acute respiratory syndrome coronavirus 2 (SARS-CoV-2), and the main common symptoms are persistent dry cough, fever, chills, and shortness of breath.^(1)^ In Brazil, as of June, 2021, more than 16 million people have been infected, and more than 470,000 have died as a result of this infection.^(2)^

During the 2020 COVID-19 outbreak, health authorities issued safety recommendations for all populations, including mobility restrictions and limitations for outdoor regular physical activity to control the spread of the virus.^(3,4)^ However, these recommendations may have caused a substantial reduction on physical activity levels. Regular and moderate/vigorous physical activity is known to reduce risk for all-cause mortality and improve immune function.^(5,6)^

People with obesity are more likely to be physically inactive compared to those with normal weight (body mass index 18.5-24.9kg/m^2^).^(7)^ In addition, overweight/obesity has been associated with increased risks of biological health-related complications by COVID-19.^(8)^ Of note, excessive fatness threatens the immune system and reduces the body’s acute and adaptive responses to infections.^(9,10)^ This population is classified as high-risk, and the stay-at-home orders are stricter for people with obesity.^(11)^ It is important to mention that women and men had different reactions to quarantine.^(12)^Women were more susceptive to the deleterious effects of stay-at-home order and social isolation, showing more feelings of anxiety, depression, low self-esteem, sadness, and stress.^(13)^ Thus, it is plausible to speculate that people with obesity are also more likely to suffer deleterious effects of living through a pandemic and social isolation on physical activity levels, and this effects may be mediate, at least partially, by the sex of the person.

The hypothesis of the present study is that individuals with overweight/obesity will present lower levels of physical activity when compared with those with normal weight.

## OBJECTIVE

To verify the association of changes on physical activity levels during COVID-19 outbreak of individuals with normal weight and overweight/obesity, and the influence of sex in this relationship.

## METHODS

### Study design, setting, and participants

This cross-sectional study was conducted in line with the Strengthening the Reporting of Observational Studies in Epidemiology (STROBE) checklist, to ensure the quality of the present study. A survey was conducted in Brazil between May 5 and May 17, 2020. Participants were invited to volunteer through social media (Facebook, Twitter, Instagram, and WhatsApp) to answer a structured electronic questionnaire. This study aimed to investigate an adult population, therefore, only the surveys of participants over 18 years of age were considered for analysis.

Inclusion criteria were participants with 18 years of age or older that answered all the questions within the survey.

A questionnaire (in Portuguese) via Google Forms was presented to participants with 70 questions divided into seven domains: personal information; COVID-19 personal care; physical activity; eating behavior; health risk habits; mental health and overall health. The instrument was developed by senior researchers with PhD in different areas (Public Health, Science, Nutrition, Physiology, Human Movement Science, Neuroscience and Behavior). Questions related to the COVID-19 pandemic were created based on face validity. For the purpose of the present study, only some questions of some domains were selected and analyzed, such as personal information, COVID-19 personal care, physical activity, and overall health. The questions used in the present analysis will be discussed later.

### Variables

#### Dependent variable

Impact of COVID-19 in physical activity practice. The impact of COVID-19 on physical activity level was assessed through the question: “How much has the COVID-19 pandemic affected your daily physical activity habits?”. The subjects were grouped into the following categories: “No” (if their response was either “none” or “a little”) and “Yes” (if they indicated “a lot”).

## Independent variable

### Overweight definition

Height and weight were self-reported (overall health domain) through the questions: “What is your weight (in kilograms)?” and “What is your height (in centimeters)?”. The values were used for standard body mass index (BMI) calculation (kg/m^2^). Following the WHO recommendations,^(13)^ overweight was defined as BMI ≥25kg/m^2^.

## Possible confounders

### Personal information

From this domain, self-reported information including sex (possible answers: woman or man), date of birth (DD/MM/YYYY), and educational level (open response) was collected.

## COVID-19 personal care

The question “How long have you been in social isolation (in days)?” was included as an open response question.

### Current physical activity questions included

How many times have you been exercising a week? (possible answers: none, 1, 2, 3, 4, 5, 6, 7); How long have you exercising daily? (possible answers: none, less than 30 minutes, between 30 to 60 minutes, more than 60 minutes); and How intense is your physical activity? Low: standing work, slight household chores; Medium: walking and other moderate activities; High: jogging, running, or other intense activities (possible answers: low, medium/moderate, high, I am not exercising).

Based on these responses, time spent during each exercise session throughout the study was multiplied by the number of days spent exercising each week. Those that reached 150 minutes or more of moderate to vigorous physical activity per week were considered, “physically active,” while those below this level were classified as “inactive.”

## Overall health

This domain assessed the presence of diagnosed diseases. From the list of diseases, the participant was asked to check all that apply (possible answers: hypertension, diabetes, high cholesterol, high triglycerides, depression, arthritis/osteoarthritis/rheumatism, asthma, cardiopathy, or other).

## Statistical analysis

The sample characteristics are presented as mean and standard deviation and frequency. We used the χ^2^ test to verify the intensity of physical activity practice (moderate/vigorous physical activity) considering the sex of the sample. The comparison of moderate/vigorous physical activity (MVPA) minutes in participants with normal weight and overweight during the week was analyzed by covariance analysis (ANCOVA), adjusted for previous physical activity, age, education level, and social isolation. The impact of COVID-19 on physical activity and the association with nutritional status was analyzed using binary logistic regression considering the following adjustments: age, education level, social isolation, previous physical activity. The statistical significance used was 5% and the confidence interval adopted was 95% (p≤0.05). All statistical analyses were performed using the SPSS/PASW version 20 (IBM Corp, New York, USA).

## Ethics

This study was approved by the ethics committee of the institution *Universidade Nove de Julho* before data collection (CAAE: 30890220.4.0000.5511, opinion # 4.002.943, approved on May 1, 2020). Prior to the survey, participants were asked to sign an informed consent form. Data were collected anonymously and only those that provided consent were included in the analysis. All procedures followed national legislation and the Declaration of Helsinki.

## RESULTS

Participants consisted of 1,828 adults (58% women) and the overall prevalence of overweight/obesity was 50.1%. Table 1 shows that the prevalence of overweight/obesity in men was higher than in women, 65.5% and 39.8%, respectively (p value <0.001).

**Table 1:** Sample characterization and level of physical activity in Brazilian women and men during COVID-19 pandemic

Sex	Men	Women	

	(n=766)	(n=1,062)	p value
Age (years)	38.8 (±14.0)	36.9 (±12.5)	0.003
Weight (kg)	83.80 (±15.28)	65.80 (±12.25)	<0.001
Height (cm)	176.14 (±6.89)	163.18 (±6.35)	<0.001
BMI (kg/m^2^)	26.95 (±4.36)	24.70 (±4.33)	<0.001
	Frequency (95CI%)	Frequency (95CI%)	
MVPA (%)	33.4 (±30.1-36.7)	25.6 (±23.0-28.2)	<0.001
In social isolation (%)	94.3 (±92.6-95.9)	96.5 (±95.4-97.6)	0.029

Data presented as mean (standard deviation) or relative frequency (95% confidence interval).

BMI: body mass index; 95%CI: 95% confidence interval; MVPA; moderate to vigorous physical activity.

Table 2 shows the results related to MVPA during COVID-19. Overall participants and women with normal weight spent more minutes in MVPA compared to overweight group (p<0.001).

**Table 2:** Mean time of moderate/vigorous physical activity during COVID-19 pandemic

	Minutes in MVPA/week		

	Mean	SE	p value
Total sample			
Normal weight	98.97	3.08	
Overweight/obesity	84.60	3.06	<0.001
Men			
Normal weight	110.13	6.10	
Overweight/obesity	97.54	4.41	0.103
Women			
Normal weight	92.49	3.46	
Overweight/obesity	72.13	4.27	<0.001

Adjusted by age, education level, and social isolation.

MVPA: moderate/vigorous physical activity; SE: standard error.

Table 3 shows the association of the impact of COVID-19 on physical activity levels and BMI, stratified by sex. Associations were found between women and men with overweight/obesity and impact of COVID-19 on the physical activity practice compared to normal weight, adjusted by age, education level, social isolation, and previous physical activity.

**Table 3:** Association of the impact of COVID-19 on physical activity levels and body weight classification in Brazilian women and men

	Impact of COVID-19 in PA practice		

	Odds ratio	95%CI	p value
Total sample			
Normal weight	1.00	1.00	
Overweight/obesity	1.57	1.26-1.94	0.001
Men			
Normal weight	1.00	1.00	
Overweight/obesity	1.53	1.07-2.17	0.017
Women			
Normal weight	1.00	1.00	
Overweight/obesity	1.67	1.25-2.22	0.001

Model adjusted by age, education level, social isolation, previous physical activity.

PA: physical activity; 95%CI: 95% confidence interval.

## DISCUSSION

The main finding of the present study was that during the COVID-19 outbreak, women and men with overweight/obesity are more likely to perceive changes in physical activity when compared with normal weight counterparts. Quarantine affected body weight gain (around 2.2-4.4kg), indicating decreased physical activity as one of the main risk factors.^(14)^ In line with previous research examining physical activity during COVID-19 crisis, all levels of physical activity decreased, while sitting or screen time increased.^(15,16)^ However, no studies included nutritional status in their analysis. Few studies found opposite results, showing an increase of physical activity during COVID-19 crisis.^(16,17)^ These differences may be related to different governmental policy on movement restrictions during this period. Of note, in an Australian population it was observed that 43.4% of the population (n=5,469) exercised less during the COVID-19 pandemic and found a relationship between binge eating and physical activity.^(14)^

The present research highlights the potential impact that COVID-19 stay-at-home orders and social isolation have had on physical activity among Brazilian adults, in addition to identifying groups that may be most in need of support. Social isolation measures are likely to have wide ranging effects that make physical activity behaviors more difficult for many, and there have been several suggestions that social restrictions to limit the spread of COVID-19 may result in population level weight gain.^(18,19)^ The findings of the present study support this suggestion and highlight that adults with higher BMI may be most at risk to have lower physical activity levels, and consequently increased weight gain as a result of the COVID-19 crisis. In addition of being an established risk factor for all-cause mortality, obesity is now also thought to be a risk factor for COVID-19 mortality.^(20,21)^

In practical terms, besides the positive effects of the social isolation to limit the spread of infection, in adults with obesity, prolonged home stays can increase physical inactivity. In addition to changes in immunologic function, social isolation can contribute to anxiety and depression, and inappropriate food habits in this specific group.^(22,23)^ Global action supporting healthy diet and physical activity is mandatory to encourage people to return to a healthy lifestyle. This action needs to be stronger for individuals of a low socio-economic level that will suffer to a higher degree from the inevitable restrictions and economic crisis following a vast and prolonged quarantine.^(18)^ In this context, remote exercise strategies such as supervised home-based exercise programs could help to attenuate the consequences of social isolation, since these programs can increase physical activity levels and potentially have a positive effect on mental health.^(24)^

The main limitations of this study include the lack of objective measurement of body mass, physical activity, and the reliability and validity of self-report responses. However, this is the first study specifically related to COVID-19, which worked with a sample including normal weight, and overweight/obese individuals.

## CONCLUSION

The results of this study showed an association between weight and changes in physical activity levels, even when controlled for previous physical activity levels. Individuals with overweight/obesity were more likely to reduce their physical activity levels during COVID-19 pandemic, regardless of sex. Strategies to improve physical activity in this specific group (overweight/obesity) should be developed to minimize the long-terms consequences of COVID-19 and physical inactivity in their psychophysiological health.
